# Ion channels as part of macromolecular multiprotein complexes: Clinical significance

**DOI:** 10.1007/s00399-017-0542-y

**Published:** 2017-12-06

**Authors:** Jordi Heijman, Dobromir Dobrev

**Affiliations:** 1 Department of Cardiology, Cardiovascular Research Institute Maastricht, Faculty of Health, Medicine, and Life Sciences, Maastricht University, 616, 6200 Maastricht, The Netherlands; 2 Institute of Pharmacology, West German Heart and Vascular Center, Faculty of Medicine, University Duisburg-Essen, Hufelandstr. 55, 45122 Essen, Germany

**Keywords:** Cardiac arrhythmias, Calcium, Electrophysiology, Macromolecular complex, Phosphorylation, Herzrhythmusstörungen, Kalzium, Elektrophysiologie, Makromolekularer Komplex, Phosphorylierung

## Abstract

Ion channels and Ca^2+^-handling proteins involved in the regulation of cardiac electrophysiology and contractility are organized in macromolecular multiprotein complexes. Recent molecular and cellular studies have significantly enhanced our understanding of the composition of these macromolecular complexes and have helped to elucidate their role in the dynamic regulation of ion channel function. Moreover, it has become clear that alterations in the composition of ion channel macromolecular complexes, for example, due to genetic mutations or acquired alterations in the expression of individual components, may lead to ion channel dysfunction and arrhythmogenesis. Here, we review novel insights into the composition of the major ion channel macromolecular complexes and discuss the potential clinical significance of alterations in these dynamic multiprotein structures.

## Zusammenfassung

Die physiologische Funktion von Ionenkanälen erfordert eine präzise Regulation, die zum Teil über die Bildung makromolekularer Multiproteinkomplexe gewährleistet wird. Studien auf zellulärer und molekularer Ebene konnten unser Verständnis über die dynamischen Regulationsmechanismen der Ionenkanalkomplexe deutlich verbessern. Es ist inzwischen etabliert, dass genetische Mutationen oder erworbene Störungen in der Expression oder Funktion der beteiligten Komplexpartner zu Ionenkanaldysfunktionen und zum Auftreten von lebensbedrohlichen Arrhythmien führen können. In diesem Übersichtsartikel fassen wir die Regulation der makromolekularen Multiproteinkomplexe ausgewählter Ionenkanäle zusammen und diskutieren die möglichen klinischen Folgen von Veränderungen in der Funktion oder Zusammensetzung dieser dynamischen Multiproteinstrukturen.

## Introduction

Every heartbeat is orchestrated by a cascade of electrical activity that initiates contraction in cardiomyocytes through a process termed excitation–contraction coupling [1, 2]. The electrophysiological properties of cardiomyocytes are dynamically regulated to adapt to varying demands. Research performed during the past 20 years has shown that the ion channels and Ca^2+^-handling proteins that are essential for cardiomyocyte electrophysiology and excitation–contraction coupling are organized in large macromolecular multiprotein complexes [3]. In these macromolecular complexes, numerous proteins interact to form a functional unit, such as an ion channel. Moreover, the precise composition of the macromolecular complexes in the cell membrane critically controls the dynamic regulation of ion channel function. As such, alterations in the composition of these macromolecular complexes can promote ion channel dysfunction with subsequent arrhythmias in various cardiovascular diseases. After a brief introduction of cardiac cellular electrophysiology, we describe the major general components of ion channel macromolecular complexes in the heart. Finally, we highlight the potential clinical relevance of alterations in the qualitative and quantitative composition of macromolecular ion channel complexes.

## Cardiac cellular electrophysiology and arrhythmogenesis

**Fig. 1:**
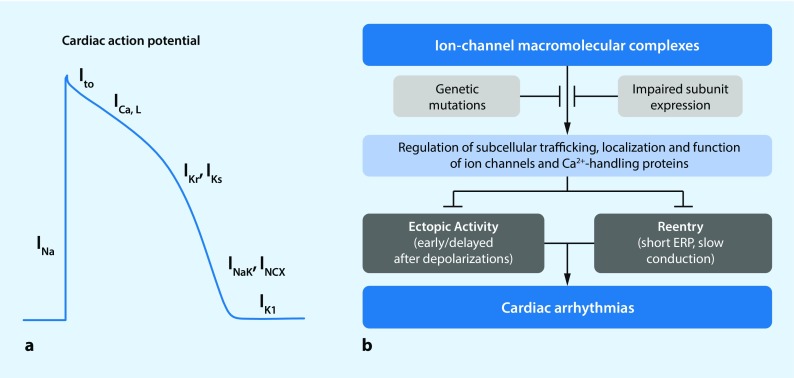
The role of ion channel macromolecular complexes in cardiac electrophysiology and arrhythmogenesis. **a** Schematic representation of the cardiac action potential and the major underlying ion currents. *I*_*Na*_  fast Na^+^ current, *I*_*to*_  transient-outward K^+^ current, *I*_*Ca,L*_  L-type Ca^2+^ current, *I*_*Kr*_  rapid delayed-rectifier K^+^ current, *I*_*Ks*_  slow delayed-rectifier K^+^ current, *I*_*NaK*_  Na^+^-K^+^-ATPase current, I_*NCX*_  Na^+^/Ca^2+^ exchanger current; *I*_*K1*_  basal inward-rectifier K^+^ current. **b** Macromolecular complexes ensure proper trafficking, localization and function of ion channels, thereby preventing occurrence of ectopic activity and reentry, the primary mechanisms of cardiac arrhythmias. This regulation may be disturbed by genetic mutations or impaired expression of individual components of the macromolecular complex. *ERP* effective refractory period

Although there are important quantitative differences in cellular electrophysiology and Ca^2+^ handling between different cardiac regions (reviewed in [1, 2, 4]), a number of commonalities and general mechanisms can be highlighted. The upstroke of the action potential (AP) in cardiomyocytes is mediated by Na^+^ influx through voltage-dependent Na^+^ channels. The resulting depolarization of the membrane potential subsequently activates several other voltage-dependent ion channels. Activation of L‑type Ca^2+^ channels produces a depolarizing inward current that shapes the plateau phase of the AP, whereas a range of K^+^ channels with different kinetic properties (e. g., slow, rapid or ultrarapid activation) control repolarization [2]. Finally, the resting membrane potential is largely determined by inward-rectifier and background K^+^ currents (Fig. 1a).

The influx of Ca^2+^ into the cardiomyocyte through L‑type Ca^2+^ channels also triggers a much larger Ca^2+^ release from the intracellular stores of the sarcoplasmic reticulum (SR) through type 2 ryanodine receptor (RyR2) channels, giving rise to the systolic Ca^2+^ transient that activates the contractile machinery [1]. Relaxation occurs when Ca^2+^ is taken back up into the SR via the SR Ca^2+^-ATPase type 2a (SERCA2a) and transported out of the cell via the Na^+^/Ca^2+^ exchanger type 1 (NCX1). Finally, homeostasis of Na^+^ and K^+^ concentrations is achieved via the Na^+^-K^+^-ATPase.

**Fig. 2:**
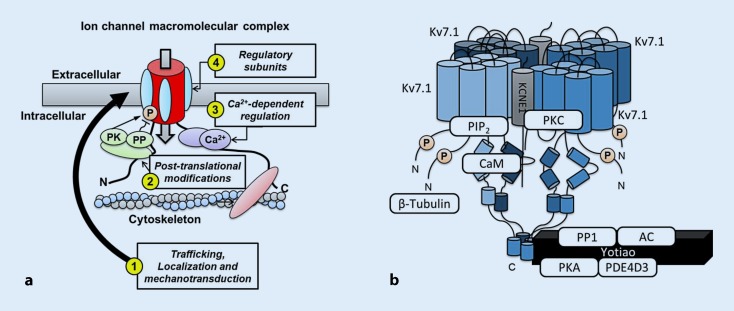
Regulation of ion channels and sarcoplasmic reticulum (SR) Ca^2+^-handling proteins through macromolecular complexes.** a** The composition of the macromolecular complex controls: *1* Trafficking, localization and mechanosensitive regulation of ion channels and Ca^2+^-handling proteins, e. g., through regulatory subunits (*cyan ellipses*) that mask endoplasmic reticulum retention signals or proteins interacting with various components of the cytoskeleton (*red*); *2* Posttranslational modifications, e. g., phosphorylation (*orange “P”*) through anchoring of protein kinases (*PK*), phosphatases (*PP*) and other enzymes (*green*); *3* Ca^2+^-dependent regulation, e. g., mediated by calmodulin (*purple*); and *4* Channel gating and biophysical properties, e. g., through regulatory subunits. **b** Example of the macromolecular complex controlling the slow delayed-rectifier K^+^ current (*I*_*Ks*_). The channel consists of a tetramer of *Kv7.1* pore-forming α subunits, *KCNE1* β subunits, an A‑kinase anchoring protein (*Yotiao*) that targets protein kinase A (*PKA*), protein phosphatase type-1 (*PP1*), adenylyl cyclase (*AC*), and phosphodiesterase (*PDE*) 4D3 to the channel, as well as the Ca^2+^-binding protein calmodulin (*CaM*), protein kinase C (*PKC*), phosphatidylinositol 4,5-bisphosphate (*PIP*_*2*_) and β‑tubulin

Each of these ion channels is in fact a large macromolecular complex consisting of numerous proteins that regulate the intracellular movement and distribution (a processes termed trafficking) and function of these channels. Dysfunction of any of these ion channels in the setting of cardiovascular disease may predispose to atrial or ventricular arrhythmias by promoting triggered activity or reentry; the major conceptual arrhythmogenic mechanisms (Fig. 1b; [2, 5]). In particular, loss of function of repolarizing currents may produce excessive AP prolongation, giving rise to early afterdepolarizations that may lead to triggered activity. Triggered activity is also promoted by Ca^2+^-handling abnormalities activating NCX1, resulting in transient inward currents and delayed afterdepolarizations. On the other hand, shortening of repolarization (e. g., due to an increase in K^+^ currents) or slowing of conduction velocity (e. g., due to loss of Na^+^ current [I_Na_] or impaired intercellular communication) increases the likelihood of reentrant arrhythmias. Studies in animal models and human samples performed over the last 20 years have provided important information about the role of different ion channel macromolecular complexes in these fundamental mechanisms of cardiac arrhythmogenesis [5].

## Common components of macromolecular complexes

A detailed description of the composition of individual macromolecular complexes of ion channels and Ca^2+^ transport mechanisms has been given in a number of review articles [3, 6–8]. Here, we provide a more general overview of the different types of proteins and their roles in ion channel (dys)function. These roles are also summarized in Fig. 2a.

### Regulatory subunits

In most ion channels, one protein (e. g., in the case of Na^+^ channels and L‑type Ca^2+^ channels) or a multimer of one or more proteins (e. g., a homotetramer of Kv7.1 proteins in the slow delayed-rectifier K^+^ channel) form the primary pore-forming α subunit through which ions cross the cell membrane. In most cases, the pore-forming α subunit interacts with several regulatory subunits that further regulate ion channel function. For example, interaction between the pore-forming Kv7.1 subunit and KCNE1 β subunits is required to produce the characteristic slow activation kinetics of the slow delayed-rectifier K^+^ current (I_Ks_) that contributes to repolarization of the AP (Fig. 2b; [9]). Most other ion channels similarly associate with different β subunits [3, 7]. Interestingly, however, the exact stoichiometry between pore-forming α subunits and regulatory β subunits remains a topic of debate for most ion channels [3, 8]. Besides classical transmembrane β subunits such as KCNE1, a wide range of other interacting proteins, binding to other parts of the pore-forming α subunit, regulate the function of ion channels and Ca^2+^-handling proteins. For example, at least a dozen regulatory subunits have been identified as part of the RyR2 macromolecular complex [1, 10], including FK-506 binding protein 12.6 (FKBP12.6), which enhances cooperation between the four pore-forming α subunits, regulating the stability of the channel’s closed state. In addition, FKBP12.6 plays a role in coupled-gating between different RyR2 channels. This process, whereby two or more connected RyR2 channels can gate simultaneously, is critical for the rapid synchronous initiation of SR Ca^2+^ release and excitation–contraction coupling [10].

### Components involved in trafficking, subcellular localization, and degradation

The number of ion channels on the membrane of a cardiomyocyte that determine the cardiac AP is dynamically regulated via trafficking, degradation, and recycling pathways [11, 12]. For several ion channels it has been established that the composition of the macromolecular complex plays a major role in their trafficking. For example, for I_Ks_, Na^+^ and L‑type Ca^2+^ channels the interaction with regulatory subunits strongly promotes forward trafficking by masking signals present in their α subunits that normally ensure that these subunits get retained in subcellular compartments (so-called endoplasmic reticulum retention signals). Thus, the interaction with regulatory subunits increases the number of functional channels in the plasma membrane [12]. In agreement with this important role in trafficking, transmural differences in the expression of the KChIP2 regulatory protein determine the gradient in transient-outward K^+^ current I_to_ from epicardial to endocardial layers of the heart [8, 13]. Interestingly, recent evidence has indicated that the composition of the macromolecular complex not only facilitates trafficking, but also determines where exactly these ion channels traffic. Interactions with different components of the actin or microtubule cytoskeleton appear to play a critical role in this targeting [14]. For example, the last three residues of the Na^+^ channel α subunit form a PDZ-binding motif that enables interaction with syntrophin and SAP97 proteins, which is required for expression of the Na^+^ channel macromolecular complex at the lateral membrane, but not at the intercalated disk [15]. Correct targeting of individual macromolecular complexes is necessary for their correct functioning. For example, plasma membrane targeting of Ca^2+^ channel Ca_v_1.3 α subunits requires direct interaction with the multifunctional adapter protein ankyrin-B to ensure proper Ca^2+^ channel function [16]. Also, the Ca^2+^-induced Ca^2+^ release from the SR requires a close interaction between RyR2 and L‑type Ca^2+^ channels [1]. This interaction depends in part on junctophilin 2 proteins that interact with RyR2 and the T‑tubular membrane where L‑type Ca^2+^ channels are predominantly located. Likewise, βII spectrin, an actin-associated molecule, is also required for proper RyR2 channel targeting [17]. Thus, the presence of junctophilin 2 and βII spectrin in the RyR2 macromolecular complex is critical for normal excitation–contraction coupling. Finally, interacting proteins may also determine the stability and degradation of ion channels, as has recently been shown for the L‑type Ca^2+^ channel and its interacting protein polycystin 1 [18].

### Components involved in ion channel posttranslational modifications

After trafficking to their specific subcellular location, almost all ion channels undergo various posttranslational modifications that dynamically regulate their biophysical properties [19]. Among these posttranslational modifications, phosphorylation by serine/threonine kinases and corresponding dephosphorylation by phosphatases have been most extensively studied [8, 20]. Given the relatively unspecific nature of phosphorylation the individual signaling components need to be highly localized in order to enable specific regulation of individual targets. A‑kinase anchoring proteins (AKAPs) are a family of structurally diverse proteins that bind kinases and phosphatases and anchor them to the macromolecular ion channel complexes [21]. For example, the targeting of protein kinase A (PKA) to the I_Ks_ macromolecular complex by the AKAP Yotiao is essential for the upregulation of I_Ks_ in response to β‑adrenoceptor stimulation (Fig. 2b; [9, 22]). Similarly, PKA is targeted to the RyR2 macromolecular complex via mAKAP, although the functional relevance of PKA-dependent RyR2 regulation remains a topic of debate [10, 23]. In addition to kinases and phosphatases, it has been shown that AKAPs also recruit other components of the β‑adrenoceptor signaling cascade to the vicinity of ion channels and Ca^2+^-handling proteins. For example, both Yotiao and mAKAP also bind adenylyl cyclases responsible for cAMP production and phosphodiesterases responsible for cAMP degradation, thereby expanding the fine tuning of local regulation of ion channel function [21, 22]. It is likely that the composition of the macromolecular complex similarly controls other posttranslational modifications. Indeed, caveolin-3 and syntrophin have been suggested to regulate Na^+^ channel S‑nitrosylation [7] and recent data have identified sirtuin 1 deacetylase as part of the Na^+^ channel macromolecular complex controlling acetylation-mediated changes in I_Na_ [24]. However, in general the molecular mechanisms controlling these other posttranslational modifications are less well established in the heart.

### Ca^2+^-dependent and mechanical regulation of ion channels

Given the central role of Ca^2+^ in the short- and long-term regulation of cardiac function, it is not surprising that a large number of feedback mechanisms exist that modulate the electrophysiological properties of the heart in response to changes in intracellular Ca^2+^ levels. Calmodulin is a multifunctional Ca^2+^-binding messenger protein expressed ubiquitously in all cells [25]. It is a common constituent of ion channel macromolecular complexes and plays an integral role in the Ca^2+^-dependent regulation of these channels [25]. For example, Ca^2+^ influx through L‑type Ca^2+^ channels undergoes strong autoregulation through Ca^2+^-dependent inactivation of the channel, which critically depends on the interaction of calmodulin with the C‑terminus of the pore-forming α subunit [25]. Similarly, calmodulin binds to RyR2 and stabilizes its gating [26]. Accumulating data indicate that also non-Ca^2+^ channels can undergo Ca^2+^-dependent regulation mediated by calmodulin in their macromolecular complex. These include Na^+^ channels, Ca^2+^-dependent small-conductance K^+^ channels (SK channels) and I_Ks_ channels, which enable a close bidirectional coupling between cellular electrophysiology and Ca^2+^ handling [3, 8, 25]. Besides calmodulin, other Ca^2+^-binding proteins can regulate the function of different macromolecular complexes. For example, calsequestrin 2 is a Ca^2+^ buffer located in the SR that also modulates gating of RyR2 channels in response to changes in SR Ca^2+^ concentrations [1]. Similarly, SR Ca^2+^ uptake via SERCA2a is negatively regulated by phospholamban and the interaction between SERCA2a and phospholamban is Ca^2+^ dependent [27], providing a way to improve SR Ca^2+^ uptake under conditions of high cytosolic Ca^2+^.

Cardiac electrophysiology is also regulated by mechanical forces acting on cardiomyocytes through a process termed mechano-electric feedback [28]. In addition to mechanically gated channels (e. g., stretch-activated channels), several traditional voltage-gated ion channels, including Na^+^ channels and inward-rectifier K^+^ currents, are now known to be modulated by mechanical forces [28]. For most voltage-gated ion channels the interaction with the lipid bilayer and cytoskeleton plays an essential role in their mechanical modulation [28]. Alternatively, ion channels may be regulated indirectly through mechanosensitive proteins such as polycystin 1, which increases the stability of L‑type Ca^2+^ channels in response to mechanical stretch [16]. As such, the composition of the macromolecular complex may also modulate mechanosensitive regulation. Finally, modulation of the mechanosensitive regulation of these channels by antiarrhythmic drugs, as has been identified for ranolazine and Na^+^ channels [29], may influence their antiarrhythmic efficacy.

## Clinical relevance

Numerous mutations in cardiac ion channels and Ca^2+^-handling proteins have been identified that lead to arrhythmia syndromes. Several of these mutations disrupt the macromolecular complex composition and subsequently promote dysregulation of ion channel function, contributing to their genotype–phenotype relationship [3, 30]. For example, loss-of-function mutations in the pore-forming α‑subunit Kv7.1, β‑subunit KCNE1 and the AKAP Yotiao have been identified that disrupt the I_Ks_ macromolecular complex and thereby prevent PKA-dependent phosphorylation of Kv7.1 and subsequent upregulation of I_Ks_ during sympathetic stimulation [9, 22]. Carriers of these mutations have long-QT syndrome and are at an increased risk for ventricular tachyarrhythmias, particularly during conditions of elevated sympathetic tone. Similarly, mutations in RyR2 and its interacting proteins (e. g., calmodulin, calsequestrin 2, and junctophilin 2) have been identified that disrupt the interaction with these stabilizing regulators, promoting aberrant RyR2 gating and predisposing patients to catecholaminergic polymorphic ventricular tachycardia and/or familial atrial fibrillation (AF; [1, 30, 31]). Finally, the phospholamban Arg14del mutation, which predisposes patients to a severe arrhythmogenic cardiomyopathy, produces a remarkable change in macromolecular complexes in mice by switching from the SERCA2a macromolecular complex to the Na^+^-K^+^-ATPase complex [32], highlighting the intricate regulation of macromolecular complex composition.

In addition to genetic variants that perturb protein–protein interactions, dysfunction of macromolecular complexes can result from an imbalanced expression of individual components or altered binding affinities due to cardiovascular disease (Fig. 2a). Although there is limited information about the dynamic changes in macromolecular complex composition during cardiovascular diseases in humans, recent data have provided several examples of macromolecular complex dysregulation in AF patients. For example, in paroxysmal AF patients, the expression of RyR2 was increased, but expression of junctophilin 2 was unchanged [31]. Furthermore, RyR2 single-channel activity was enhanced in the absence of increased RyR2 phosphorylation, suggesting that a relative deficiency of stabilizing junctophilin 2 in the macromolecular complex may contribute to RyR2 dysfunction and subsequent triggered activity in these patients [33]. By contrast, RyR2 dysfunction in patients with long-standing persistent AF has been attributed to RyR2 hyperphosphorylation [34] and preliminary data suggest that this might be due to an imbalance in kinase and phosphatase levels within the macromolecular complex [35]. Similarly, the increased relative amount of protein kinase C within the NCX1 macromolecular complex of long-standing persistent AF patients may contribute to the increase in NCX1 activity that also promotes proarrhythmic triggered activity in these patients [36]. Thus, both genetic and acquired changes in macromolecular complex composition may promote cardiac arrhythmias.

## Conclusions and future perspectives

The tightly controlled regulation of ion channel function plays a critical role in cardiac (patho)physiology. Macromolecular complexes are essential for this regulation by bringing together pore-forming and regulatory subunits, proteins controlling ion channel trafficking and localization, kinases, phosphatases and other components of signaling pathways, as well as proteins involved in other forms of ion channel regulation (e. g., Ca^2+^-dependent regulation). Mutations that disrupt the physiological interaction of components of the macromolecular complex or changes in the relative protein expression or binding affinities of these components can alter ion channel function and have been implicated in arrhythmogenesis. Here, we have focused on protein–protein interactions occurring within a single cardiomyocyte, but macromolecular complexes are similarly involved in cell–cell adhesion and intercellular communication, and dysfunction of these complexes may also contribute to arrhythmogenesis (e. g., in arrhythmogenic right ventricular cardiomyopathies) [37].

Although the understanding about macromolecular complexes and their clinical relevance has increased significantly in recent years, numerous topics for further research remain. For example, information about the stoichiometry and temporal variability in the composition of macromolecular complexes is limited. Similarly, the dynamic remodeling of macromolecular complexes in response to cardiovascular disease is incompletely characterized, particularly in human cardiomyocytes. Finally, there are currently almost no (therapeutic) interventions to selectively and reversibly alter the interactions between different components of a macromolecular complex. A better understanding of the composition and clinical relevance of ion channel macromolecular complexes and the subsequent development of new therapeutic options based on this knowledge is expected to enable a more effective, mechanism-based treatment of heart rhythm disorders.
